# A database of optimal integration times for Lagrangian studies of atmospheric moisture sources and sinks

**DOI:** 10.1038/s41597-019-0068-8

**Published:** 2019-05-16

**Authors:** Raquel Nieto, Luis Gimeno

**Affiliations:** Environmental Physics Laboratory (EPhysLab), CIM-UVIGO, Universidade de Vigo, Ourense, 32004 Spain

**Keywords:** Hydrology, Atmospheric science

## Abstract

Lagrangian methods for estimating sources and sinks of water vapour have increased in importance in recent years, with hundreds of publications over the past decade on this topic. Results derived from these approaches are, however, very sensitive to the integration time of the trajectories used in the analysis. The most widely used integration time is that derived from the average residence time of water vapour in the atmosphere, normally considered to be around 10 days. In this article, we propose an approach to estimate the optimal integration time for these Lagrangian methods for estimating sources and sinks, by comparing estimates of precipitation from the Lagrangian approach using different times of integration with results obtained from three state-of-the-art reanalyses, thereby providing a database of optimal integration times per month, for a spatial resolution of 0.25° × 0.25° in latitude and longitude.

## Abstract (summary)

**Table:** 

Design Type(s)	modeling and simulation objective • time series design • statistical parameter determination objective
Measurement Type(s)	parameter
Technology Type(s)	computational modeling technique
Factor Type(s)	geographic location • temporal_interval
Sample Characteristic(s)	Earth (Planet) • climate system

Machine-accessible metadata file describing the reported data (ISA-Tab format)

## Background & Summary

In any understanding of the global hydrological cycle, one of the key variables is the residence time of water vapour in the atmosphere. Studies based on dividing the atmospheric reservoir by the incoming or outgoing flux typically estimate the residence time at around 8–10 days^1^. This quantity clearly has significant variability in space and time, with marked seasonality and sharp variations with latitude and orography, and is also very dependent on weather conditions, being very short when local or mesoscale meteorological systems occur, and much longer in the upper troposphere where few precipitating systems are found. By taking W/P or W/E (where W is the water in the local atmospheric column, P is precipitation and E evaporation) Trenberth^2^ found residence times of approximately 9 days. Using moisture tracking models (semi-Lagrangian^3^ or Eulerian^4^), residence times ranged between approximately 7 days (spring) and 9 days (summer), values very close to those found in classic studies. Using age tracers and a global circulation model, Numaguti^5^ also found residence times of around 10 days, and this study has been widely cited in the scientific literature since then. For a more complete review of the different methods and results obtained, the reader is referred to the introduction of van der Ent and Tuinenburg^6^, but suffice it to say that residence time has not generally been considered a controversial variable in scientific terms until now, commonly being estimated at around 8–10 days.

More recently, the scientific literature has seen a great deal of controversy regarding the estimation of average values of residence time, which could point to the adoption of more specific values by place and season than the 8–10 day figure. Two studies, using completely different techniques, produced clearly divergent results. Using average times of the duration of the phase of the humidity gain of so-called particles with a Lagrangian method based on backward trajectories computed by the Lagrangian particle tracking method FLEXPART (FLEXible PARTicle dispersion model)^7,8^ forced with the reanalysis ERA-Interim^9^, Läderach and Sodemann^10^ obtained times as short as 4–5 days, just half the value found in previous studies. In response, van der Ent and Tuinenburg^6^ used two different atmospheric moisture tracking models (WAM-2layers and 3D-T) to obtain a residence time of around 8.5 days based on ERA-Interim data. In both papers^6,10^, spatial maps of residence time were generated.

Residence time is not only of theoretical interest in the study of the hydrological cycle; many estimates of other characteristics of the hydrological cycle also depend on it, in particular our concern here is the estimation of sources and sinks of moisture. Lagrangian methods for estimating sources and sinks have gained in importance in recent years due to the reliability of the technique, which is able to track the positions of so-called particles, and estimate the specific humidity changes experienced by them on their trajectories (see Gimeno *et al*.^11^ for a review of this topic together with advantages and disadvantages of the different techniques used to estimate moisture sources). These Lagrangian techniques have been used extensively in hundreds of studies over the past decade, the great majority using 10 days as the integration time^5^. Self-evidently, the results of these studies (of sources and sinks) are highly sensitive to the time used for integration, identifying sources closer to the target region when short times are used, approaching those found in Läderach and Sodemann^10^, and much further away when conventional times are used, as in van der Ent and Tuinenburg^6^, these being much closer to the estimates made to date.

**Fig. 1:**
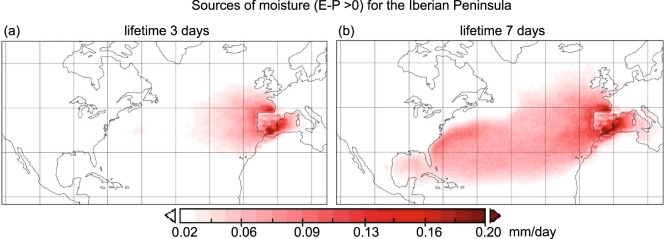
Comparing integration times for the moisture source definition for the Iberian Peninsla. (E-P) > 0 fields (in mm/day) calculated from the FLEXPART outsputs in its backward mode to determine the sources of moisture for the Iberian Peninsula, (**a**) using 3 days or (**b**) 7 days.

One example of the possible strong divergence is illustrated in Fig. 1, based on the dispersion model FLEXPART^12^ v9.0 (https://www.flexpart.eu/) and the Stohl and James (2004, 2005)^7,13^ approach for the same target region (Iberian Peninsula) and season (winter). The results differ greatly depending on whether we use an integration time of 3 days as per Läderach and Sodemann^10^, or a time of 7 days as per van der Ent and Tuinenburg^6^.

Although these Lagrangian techniques are based on estimates of Evaporation minus Precipitation, they have also been used extensively in dozens of papers, with great success, for the estimation of the precipitation originating from the humidity from each source to a given target region (for global analyses^14,15^, or for regional studies at a variety of different latitudes^16–18^).

In this article, we propose a different approach to the scientific problem. Here our objective is not to seek the residence time of the water vapour, but rather to seek the optimal time for the integration in these Lagrangian studies to estimate of moisture sources and sinks, a value that reflects the residence time without being precisely the same thing; as it was also shown for Eulerian approaches^19^. To this end, we intend to make use of the property of Lagrangian approaches of estimating the precipitation in a target region from the moisture transported from its sources of humidity. The grid-to-grid comparison of this estimated precipitation for different times of integration with the precipitation obtained from “state-of-the-art” reanalysis allows us to generate a database of optimal integration times at annual and monthly basis, with a degree of spatial resolution equivalent to the reanalysis of comparison; this can then be used as a reference for the estimation of moisture sources by Lagrangian techniques.

## Methods

To implement our Lagrangian moisture transport approach, we use data from the European Centre for Medium-Range Weather Forecasts (ECMWF) Interim Re-Analysis^9^ (hereafter ERA-I, https://www.ecmwf.int/en/forecasts/datasets/reanalysis-datasets/era-interim). This reanalysis covers the period from January 1979 to the present, and contains data at six-hourly intervals with a spatial resolution of 1° × 1° in latitude and longitude; in our study this is downscaled to 0.25 degrees using linear interpolation, for 61 vertical levels (1000 to 0.1 hPa). Comparison with other reanalyses indicates that ERA-I is the most appropriate for representing the hydrological cycle^20^.

To compare monthly precipitation estimated from the Lagrangian approach with gridded data, in addition to the monthly precipitation taken from the reanalysis ERA-I we have also used precipitation derived from two other databases, namely GPCP and MSWEP. GPCP (Global Precipitation Climatology Project, https://climatedataguide.ucar.edu/climate-data/gpcp-monthly-global-precipitation-climatology-project) is a well-known initiative of Global Energy and Water Cycle Exchanges (GEWEX) activity for more than 20 years, and has been used in thousands of papers. The GPCP version used in this study (v2.2) has a native spatial resolution of one degree of latitude-longitude, and a daily temporal resolution, and is derived from the integration of various satellite data sets over land and ocean and analysis of rain gauge data over land^21^. MSWEP (Multi-Source Weighted-Ensemble Precipitation, http://www.gloh2o.org/) is a global precipitation dataset specifically designed for hydrological modelling, which optically merges with other high quality precipitation data in a search for the best quality by timescale and location. It is available for the period 1979–2015 with a 3-hourly temporal and 0.25° spatial resolution^22^.

In this study, the data were downscaled to 0.25 degrees using linear interpolation, and aggregated over monthly intervals for the common period 1980–2015.

### A brief description of the lagrangian approach

In this study we use the Lagrangian approach^7,13^ based on the particle dispersion model FLEXPART v9.0^12^, forced by ERA-I data^9^ from the ECMWF. The atmosphere is divided into so-called particles (i.e., finite elements of volume with equal mass), and the trajectory of these particles is followed for an integration time, normally 10 days, and as commented in the introduction, this is the average residence time of water vapour in the atmosphere^5^. By summing the individual changes of specific humidity of each particle every 6 hours over a given area we can estimate the total budget of atmospheric humidity (E-P), where E denotes evaporation and P denotes precipitation. By taking all particles leaving a given source region and reaching a target region, and then selecting those particles that lose humidity in the target region, the aggregation of these losses of specific humidity for all these particles yields the transport of humidity for precipitation from the source region to the target region for a given daily, monthly, or yearly time scale. This Lagrangian method has been used many times with great success in the analysis of moisture transport^15,23^. An assessment of its advantages and disadvantages compared to other methods of tracing water vapour can be found in two recent review articles^11,24^.

### Sequence of steps to calculate optimal integration times and quality control

#### Estimation of a first approximation of the gridded precipitation calculated from the Lagrangian method (PLi) for multiple integration times

**Fig. 2:**
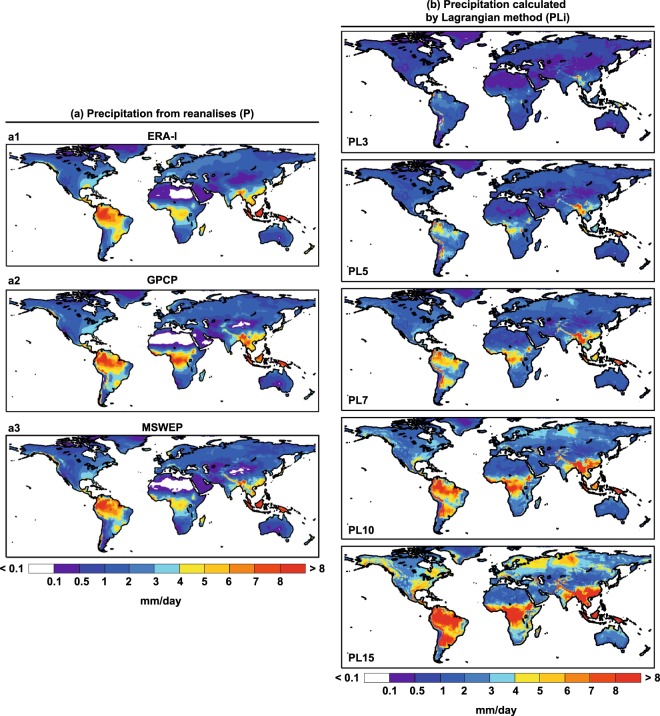
Continental precipitation from reanalysis data and those calculated the Lagrangian approach using different times of integration for the period 1980–2015. (**a**) Precipitation over continents from ERA-I (a1), GPCP (a2) and MSWEP (a3). (**b**) Continental precipitation calculated by the Lagrangian method (PLi) for 3, 5, 7, 10, and 15 days of integration over each grid element.

After dividing the world into two large sources, namely the entire continental area and the entire oceanic one, FLEXPART is run in forward mode from these two global sources, taking only the negative E-P values as in previous calculations of the contribution of moisture sources to precipitation, for the period 1980–2015. This contribution of the moisture sources to precipitation is termed “precipitation calculated by Lagrangian method” (PL), although in itself this is merely an approximation to the precipitation, being instead the contribution of moisture sources to it. In this way and for each grid element of 0.25° lat × 0.25° lon, we have two values of PL, one corresponding to the terrestrial source (PLT) and another corresponding to the oceanic source (PLO). This calculation is done for different integration times, from day 1 to day 15 (i = 1, …, 15), and we thus obtain the contribution of the oceanic and terrestrial sources to the precipitation for a range of integration days in each element of the grid (PLOi) and (PLTi). From the sum of these two quantities, we obtain a first approximation to the precipitation calculated by Lagrangian methods (PLi) for each integration time and grid element. Plots of these values are shown in Fig. 2 together with the precipitation (P) obtained from the three different reanalyses.

#### Comparison of PLi with precipitation taken from various reanalysis P and choice of optimal integration time

For a single integration time (Fig. 2), the values of precipitation obtained from the reanalyses and the precipitation calculated by Lagrangian methods may not coincide. This disagreement is the basis of this study; for example for a time of integration time of 5 days there are regions where the adjustment is good, and therefore the integration time is appropriate (e.g., Northeast Brazil), and others where agreement is poor, because the integration time of 5 days is too large, as for the precipitation linked to the Choco jet in northwestern South America^25^, or too small, as for extratropical regions with precipitation due to moisture transported long distances in the middle troposphere, as in the North Iberian Peninsula^23^.

**Fig. 3:**
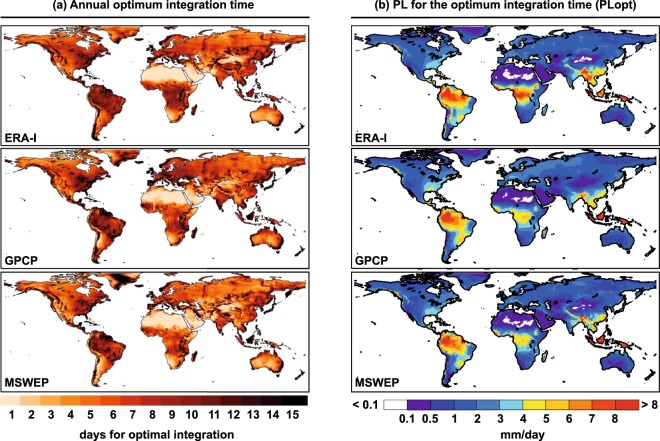
Gridded annual optimum integration times and precipitation calculated from the Lagrangian outputs for the optimum integration times (PLopt). (**a**) Annual optimum integration times from ERA-I^29^, GPCP and MSWEP. (**b**) Continental precipitation calculated by the Lagrangian method for the optimum integration time (PLopt) for each reanalysis over each grid element.

For this reason, for each grid element and integration time, PLi is compared with the precipitation obtained from different reanalysis (P), and the integration time is chosen for each grid element where the difference between these two values is as small as possible. Figure 3 shows these integration times on an annual scale together with PL for the optimum integration time (hereafter PLopt). For the three reanalyses used in the comparison, ERA-I, MSWEP and GPCP, the results are very similar for very low integration times of around 1–2 days in the desert and semi-desert regions of the African and Asian continent, for times of 3–5 days in the extratropical regions of the Southern Hemisphere, 5–7 days in the interior of North America and Eurasia, and times greater than 9 days, and as high as 15 days in regions affected by the storm track of the Northern Hemisphere (East Coast of North America, Japan, and Western Europe), and those affected by important Low Level jet systems such as North and Central South America or the Indian coasts of southern Africa and the Indian subcontinent. These integration times provide reliable preliminary results, with very good adjustments indicated by spatial correlations exceeding 0.97 for the three reanalyses.

A number of different concepts are described here; nevertheless a useful comparison can be made with the results of the average residence times of water vapour in Läderach and Sodemann^10^ (2016; their Fig. 2a) and in van der Ent and Tuinenburg^6^ (2017; their Fig. 2c). In general terms, the spatial distribution in continental regions with non-negligible precipitation is similar in all three cases, thus for example in North America the residence time is smaller in the western than in the eastern half, and the same applies for the optimal integration times. In South America, on the LLJ track from north to south through the centre of the subcontinent, the residence time is greater than it is to the east and west, and so also for the optimal integration time. Similar characteristics are seen for the inner part of the Indian Ocean coast of the African continent, and in the high values of both residence times and optimal integration times in the coastal Asian monsoonal regions. This is not seen when the precipitation is very low (such as in the North of Eurasia) or almost nonexistent, as in the desert regions of North Africa or Central Australia, where residence times are very high and the times of integration are very low. In these regions, the mechanisms in place are somewhat different. Given the low efficiency of the precipitation mechanisms and/or the low humidity, the water vapour in these regions lasts a long time, hence the high residence time, but if we track the water vapour that generates precipitation for a given event, a concept closer to our optimal integration time, this travels a shorter distance and for less time, being linked to mesoscale events such as the development of storms.

**Fig. 4:**
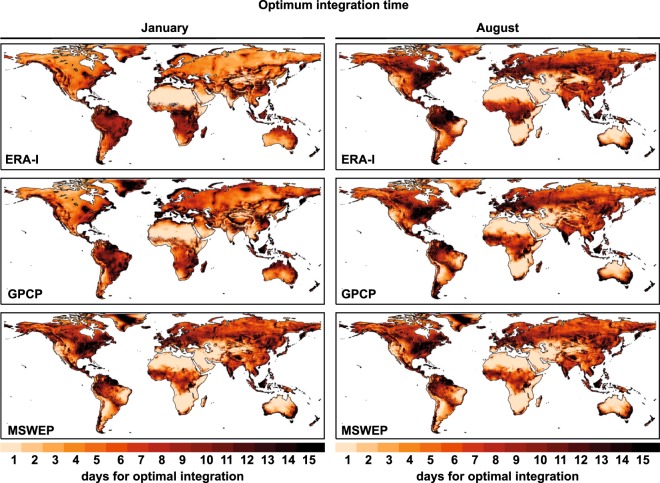
Gridded monthly optimum integration times. Optimum integration times for January and August from ERA-I^29^, GPCP and MSWEP.

Given the seasonal variations inherent in moisture transport and precipitation, it is also appropriate to perform these calculations on a monthly basis, and for this reason we present also a database of integration times by month in this paper. As an example, Fig. 4 shows values of optimal integration times for the central months of boreal and austral winter (January and August). Clear differences between these two months are apparent in all regions; for example, in extratropical regions affected by the passage of winter storms, such as the Iberian Peninsula in January, integration times are very high with a threshold of 15 days, while in summer (August), convective rainfall is much more important, giving an integration time of around 5–7 days. Of particular note are the differences that occur in the values of annual integration in regions affected by monsoon circulation or with precipitation linked to strong systems of LLJs. For example, for the west coast of the Indian subcontinent the times vary from very small (around 1–2 days) in January to 15-day periods in August. A region-by-region analysis is possible using the continental geographical regions used in the IPCC 5th Assessment Report^26,27^ (Supplementary Fig. S1), revealing the effect of changing regional weather patterns with season and the logical effect of these on the time of integration. For instance, the Sahara region is the area with the lowest values for the optimal integration time (near to 1 day). Low values are also found during summer in West and Central Asia, the Mediterranean region, South Africa or North Australia; regions with low mean summer precipitation and mainly due to convective events. In these arid or semiarid regions, the results could be related to spurious relationships in the driving data related with thermal gradients between the surrounding maritime areas and the hot land surface.

**Fig. 5:**
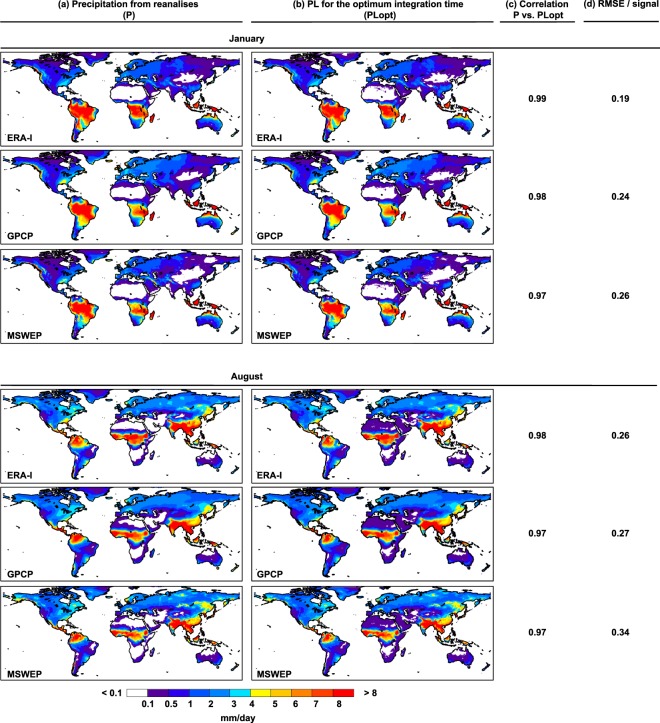
Gridded monthly precipitation from reanalysis (P) and from the Lagrangian outputs for the optimum integration times (PLopt) and their correlation. For January and for August: (**a**) precipitation from ERA-I, GPCP and MSWEP; and (**b**) precipitation calculated by the Lagrangian method for the optimum integration time (PLopt) for each reanalysis over each grid element, from ERA-I^29^, GPCP and MSWEP. (**c**) Correlation coefficient between P and PLopt. (**d**) RMSE/signal between P and PLopt.

A snapshot of the climatological values of P and PLopt for these two months (Fig. 5) shows a very good match, with excellent spatial correlations greater than 0.98, which indicates the usefulness of these integration times classified by grid element and month. In addition, the Root Mean Square Error (RMSE)^28^ by the signal (P in this case) is calculated as

1 $$\sqrt{\frac{1}{n}\,\sum _{j=1}^{n}{((PLop{t}_{j}-{P}_{j})/{P}_{j})}^{2}}$$

with n the number of grid points (n = 520000). The values indicate very low uncertainties in the mean moisture source estimation, with values around 0.2–0.3, equivalent to about 0.05% of average relative error by grid between P and PLopt.

#### On the convergence of the estimated precipitation with the precipitation obtained from reanalysis considering integration time

The sources and sinks estimated from this Lagrangian approach are dependent on the integration time, which determines the sizes of the sources and sinks. By extending the integration time in the approach used to identify the sources, these can be shown to grow endlessly and to occur exactly with the sinks. An example can be seen in Supplementary Fig. S2, in which the sources for the Iberian Peninsula in January are plotted for several integration days up to 20 days. Sources grow in both extension and integrated value (see Supplementary Fig. S3 for an example for the Iberian Peninsula) with the integration day and there is no integration day on which they are stationary. Taking the sources for each integration day as calculated in Supplementary Fig. S2 and running FLEXPART in forward mode to calculate the contribution to precipitation in January on the Iberian Peninsula from the estimated sources, it is possible to observe also that the contribution to precipitation increases with integration day in line with the sources, without converging on an asymptotic value (Supplementary Fig. S4). The optimum integration time cannot therefore be determined by identifying the day on which precipitation no longer continues to increase. In summary, because the technique in itself does not provide a convergent diagnosis of sources and sinks, it cannot be expected that the estimation of precipitation will converge with the precipitation obtained from reanalysis at a given time. That said, it should not be difficult to obtain an optimal value in which the approximation does not converge. In optimization theory, for the determination of optima we must first define an objective function, which in our case is defined as the absolute value of PL-P, with PL being an estimate of precipitation from the Lagrangian analysis, and P being the precipitation obtained from reanalysis. The necessary and sufficient condition for the existence of an optimum value is that the objective function has a maximum or a minimum in the interval of definition, and that a local optimality is thus implied. Supplementary Fig. S5 shows the values of our objective function for four grid points in the analysis (the same thing occurs for the remainder of the grid points as well) and for the months of January and August. The function has a minimum and an optimal value exists, this minimum being the one used to calculate the optimum integration time. Values close to 1 (such as those occurring during August over Northeastern Brazil and South Africa in Supplementary Fig. S5) imply that the minimum is reached at some time during day 1 (the original results were produced every 6 hours).

## Data Records

All datasets, which includes optimal integration time using ERA-I as reference reanalysis on an annual and monthly basis, are freely available at the link provided from the Zenodo repository^29^ licensed under a Creative Commons Attribution 4.0 International License (CC BY). The accompanying ‘Readme.pdf’ file provides all the necessary information concerning the format of the data and the file contents. Estimation of optimal integration times using GPCP and MSWEP base data was only done for comparison purposes and not included in the data records.

## Technical Validation

**Fig. 6:**
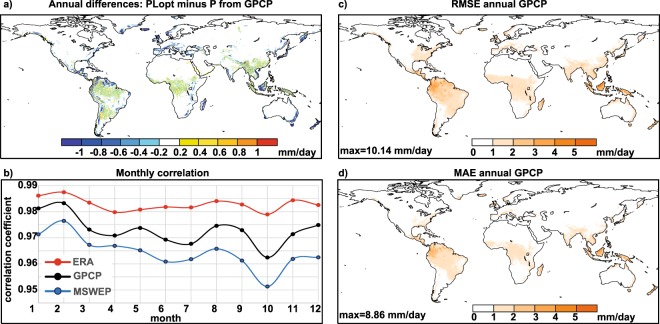
Technical validation for derived precipitation data. Technical validation for annual fields for GPCP derived precipitation data: (**a**) Difference between annual climatology of P and PLopt, (**c**) Root Mean Square Error (RMSE) and (**d**) the Mean Absolute Error (MAE). Units in mm/day. (**b**) Monthly spatial correlations of PLopt with P for ERA-I (red line), GPCP (black line) and MSWEP (blue line).

For quality control purposes, we used three different procedures. First, we calculated the difference between annual climatologies of PLopt and P (Fig. 6a for GPCP). The coincidence is high, and only negative differences appear in the continental coastal areas (FLEXPART underestimates the precipitation in some regions where the optimal integration time perhaps is also higher than 15 days, as commented previously), and positive and negative difference between ±0.4 mm/day are showed over those areas of global maxima precipitation as the Amazon, La Plata and Congo River basins, and Southeast Asia (including Mekong River). Second, we generated maps of the Root Mean Square Error (RMSE) and the Mean Absolute Error (MAE) according to GPCP, two widely used indicators of performance validation^28^. Both RMSE (Fig. 6c) and MAE (Fig. 6d) show values near to zero, indicating that both sets of values are quite similar. The major RMSE and MAE values are observed over the same areas where the annual differences are detected.

Then, we calculated the spatial correlations of PLopt with P for each month for the whole set of 36 years used (Fig. 6b). For the three reanalyses and for all months, the spatial correlations were greater than 0.95. As expected, the best correlations occurred for ERA-I because it is the input data set used to run FLEXPART and PL is therefore calculated from this, followed by GPCP and MSWEP. The month of highest spatial correlation occurs in the Northern Hemisphere winter, specifically in February with values of around 0.98 for the three reanalyses, reaching values close to 0.99 for ERA-I. The month of worst spatial correlation is October in the transition between summer and winter circulation in the Northern Hemisphere, with correlations between 0.95 for MSWEP and 0.98 for ERA-I.

**Fig. 7:**
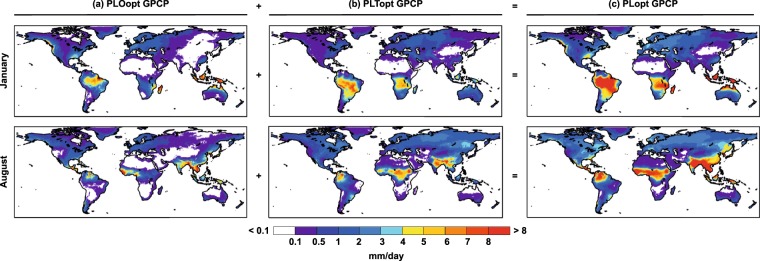
Monthly oceanic and terrestrial components of PLopt for GPCP. Precipitation according to the origin of humidity with: (**a**) a oceanic (PLOopt) or (**b**) a continental (PLTopt) origin for the optimal integration time according to GPCP for January and August. (**c**) Sum of PLOopt and PLTopt (PLopt). Units in mm/day.

Finally, and making use of the approximation that allows us to distinguish precipitation according to the origin of humidity in continents (PLTopt) or in the ocean (PLOopt), we calculated the ratio of PLTopt to PLopt, which gives an idea of the recycling and allows a comparison with two previous studies using a similar ratio but with different methodologies. Figure 7 shows PLopt, PLTopt, and PLOopt for the months of January and August, using the optimal integration time calculated by comparison with precipitation according to GPCP. This plot is interesting in itself, and leads to a discussion of the results linked to meteorological and precipitation patterns known as the higher precipitation of continental origin, as compared with precipitation of oceanic origin, for the Amazon^30^ and Congo^31^ basins in January or their seasonal variations, but this is somewhat beyond the scope of this paper. Here these plots are used to illustrate the potential of the method and how our results can be compared with those obtained using other methodologies.

**Fig. 8:**
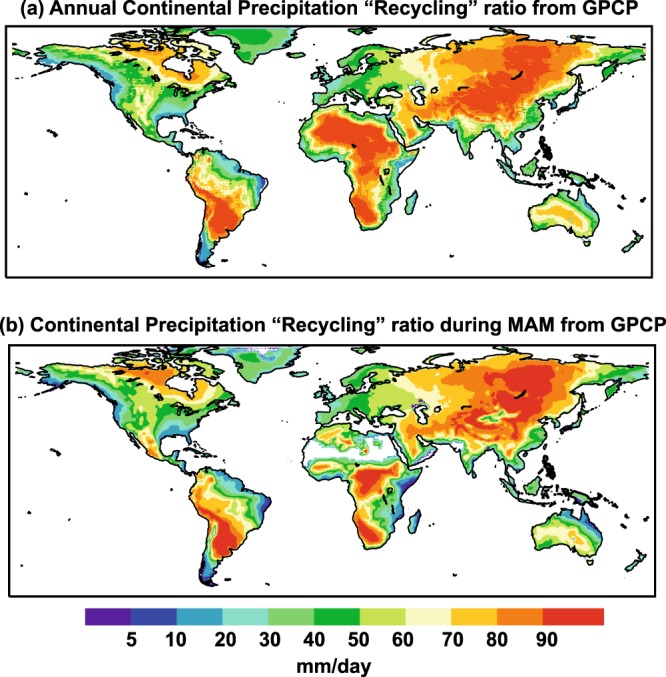
Continental precipitation recycling ratio from GPCP calculated from the FLEXPART run. Ratio of PLTopt by PLopt that approximates to the precipitation with a terrestrial origin (namely “recycling”) from results modelled by the outputs of FLEXPART using GPCP reanalysis. (**a**) Annual recycling, and (**b**) for March, April and May.

Dividing PLTopt by PLopt yields an approximation to the precipitation of terrestrial origin (“recycling”) as calculated in other studies. Thus Fig. 8 shows a visual comparison of the joint results for March, April and May to allow comparison with the plot of Dirmeyer *et al*.^32^, and the annual combination to allow comparison with that of Van der Ent *et al*.^33^. Despite the predictable logical differences due to the way the different methods are applied, the patterns are clearly recognisable and identifiable, with a similar distribution of maxima and minima, and comparable values.

### Applicability of the database and implications for future studies

This database is designed to allow better integration times than the generic 10-days usually taken in Lagrangian moisture transport analyses used to identify moisture sources and sinks. Although specific for a Lagrangian approach, that based on Stohl and James^7,13^ one, which is the most widely used with more than 70% of the papers to determine sources and sinks in the last semi-decade, it could be applicable to other similar Lagrangian approaches, whose results are very similar to those reached with the used approach when the same integration time is taken.

However, the method also allows a distinction to be made between precipitation originating from global terrestrial and oceanic sources, with the same resolution of 0.25°. Being gridded with a high spatial resolution as well as a temporal scale that could be higher than monthly, these data show great potential to differentiate at global scale terrestrial-versus-oceanic components for any region of interest (tropical forests, regions of strong precipitation deficits, hydrological basins…) and advance and complement the results of other methods. Knowing the terrestrial-versus-oceanic component of the precipitation associated with extreme precipitation events causing droughts and floods could help advance our understanding of the role of any change in the terrestrial sources of humidity associated with deforestation, or estimating at a global scale the percentage of moisture of oceanic origin associated with the main mechanisms of moisture transport such as low level jets or atmospheric rivers^29^. A better precision for particular extreme months or for individual events the optimal integration time could be calculated following the same methodology. Likewise, these results could allow us to calculate the interannual variability and trends of these two components of precipitation, oceanic or terrestrial, in support of the interpretation of the origin of the interannual variability and the trends in global and regional precipitation detected over the last few decades^34^, with implications for the interpretation of the results of predictions of precipitation in future climates.

## Supplementary Information

### ISA-Tab metadata file

Download metadata file

### Supplementary information

Supplementary Information

## Data Availability

The code that support the findings of this study are available on request from the corresponding author [RN].
