# Addendum: A database of optimal integration times for Lagrangian studies of atmospheric moisture sources and sinks

**DOI:** 10.1038/s41597-021-00902-1

**Published:** 2021-05-07

**Authors:** Raquel Nieto, Luis Gimeno

**Affiliations:** Environmental Physics Laboratory (EPhysLab), CIM-UVIGO, Universidade de Vigo, Ourense, 32004 Spain

**Keywords:** Hydrology, Atmospheric science

Addendum to: *Scientific Data* 10.1038/s41597-019-0068-8, published online 16 May 2019

The original dataset^1^ provides spatialised climatological values of the annual and monthly Optimal Integration Times (OPT) for Lagrangian Studies of Atmospheric Moisture Sources and Sinks for the period 1980–2015. The data provide more appropriate times in days to use in Lagrangian approaches for estimating the precipitation in terrestrial target regions from the moisture transported from its sources of humidity; enabling to make a distinction between precipitation originating from global terrestrial and oceanic sources^2^.

The database provided in the original version (Version 1) can be used for general climatological studies, but has inherent limitations to higher precision analysis for any particular month. To provide a more widespread temporal coverage as required for some studies, the updated second version (Version 2) of the dataset has been calculated month-by-month for the longest period available in ERA-Interim reanalysis, from 1980 to 2018. To achieve the new individual monthly dataset, the method is the same as described in Nieto & Gimeno^2^ but it is here adjusted to each individual month along the study period (468 months). Here we present a complete dataset with a spatial resolution of 0.25º × 0.25º in latitude and longitude.

Both the original^1^ and updated^3^ datasets are freely available and hosted at Zenodo under a Creative Commons Attribution 4.0 International License (CC BY). The accompanying ‘Readme.pdf’ file provides all the necessary information concerning the format of the data and the netCDF files content.

## References

[1] Nieto R, Gimeno LP (2018). Zenodo,.

[2] Nieto R, Gimeno L (2019). A database of optimal integration times for Lagrangian studies of atmospheric moisture sources and sinks. Scientific Data.

[3] Nieto R, Gimeno LP (2021). Zenodo,.

