# Supplementary material for: A database of optimal integration times for Lagrangian studies of atmospheric moisture sources and sinks
Source: Sci Data. 2019 May 16;6:59. doi: 10.1038/s41597-019-0068-8 (PMC6522491; doi:10.1038/s41597-019-0068-8)
Supplement: Supplementary file 2 — Supplementary Information [file 41597_2019_68_MOESM2_ESM.docx]

**SUPPLEMENTARY MATERIAL:**

| **5 FIGURES** | |
| --- | --- |
| **Figure S1.** | **Mean optimum integration times.** Annual, January and august optimum integration times in days for each continental geographical regions used in the IPCC 5th Assessment Report from ERA-I. |
| **Figure S2.** | **Moisture sources for the Iberian Peninsula.** January (E − P)>0 pattern for the Iberian Peninsula based on the backward in time experiment during 20 days. Period: 1980–2016. Highlighted in red i13 denote the mean optimal time obtained for January over the region under study. |
| **Figure S3.** | **Growing accumulated evaporation values over the moisture sources.** Accumulated (E − P)>0 values (evaporation) over each moisture source (Supplementary Figure S2) for every integration times for January for the Iberian Peninsula based on the backward in time experiment during 20 days. Period: 1980–2016. Highlighted with a red dot is the mean optimal time (i13) obtained for January over the region under study. |
| **Figure S4.** | **Growing accumulated precipitation values along different days of integration.** Accumulated (E − P)<0 values (precipitation) over the Iberian Peninsula for January from each moisture source (from Figure S1) for every integration times based on the forward experiment during 20 days. Period: 1980–2016. Highlighted with a red dot is the mean optimal time (i13) obtained for January over the region under study. |
| **Figure S5.** | **Precipitation values for different days of integration.** Example of the precipitation values during the different days of integration for four grid points randomly selected for January and August. |


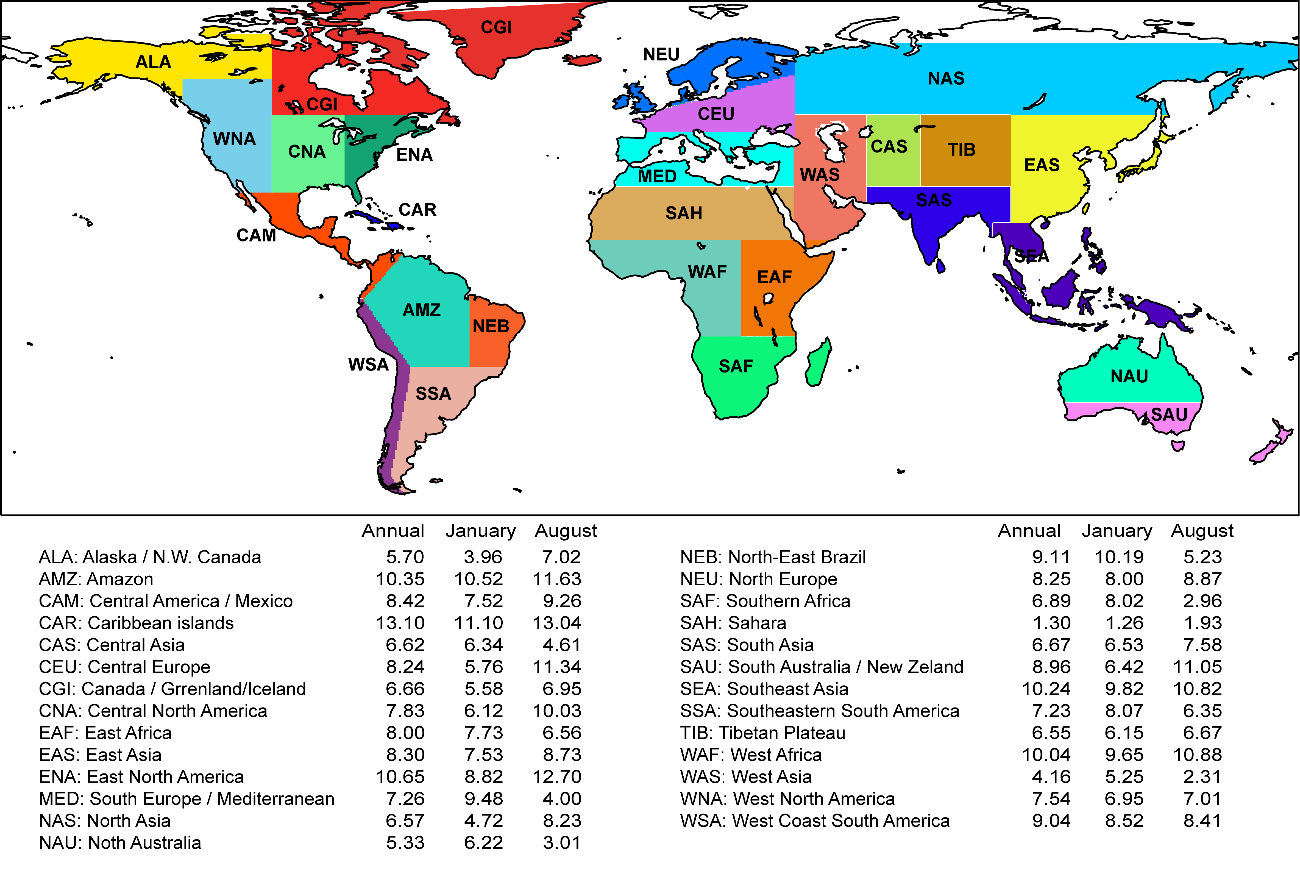


**Figure S1. Mean optimum integration times.** Annual, January and august optimum integration times in days for each continental geographical regions used in the IPCC 5th Assessment Report from ERA-I.

**
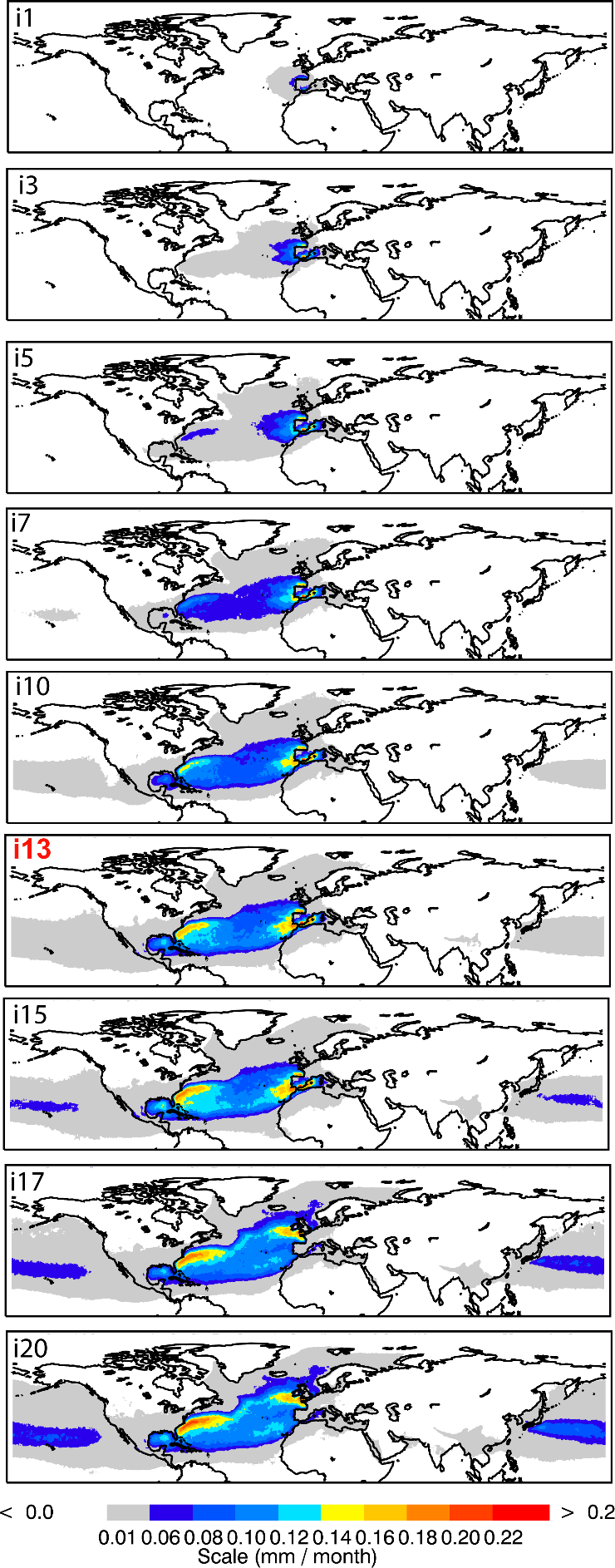
**

**Figure S2. Moisture sources for the Iberian Peninsula.** January (E − P)>0 pattern for the Iberian Peninsula based on the backward in time experiment during 20 days. Period: 1980–2016. Highlighted in red i13 denote the mean optimal time obtained for January over the region under study.


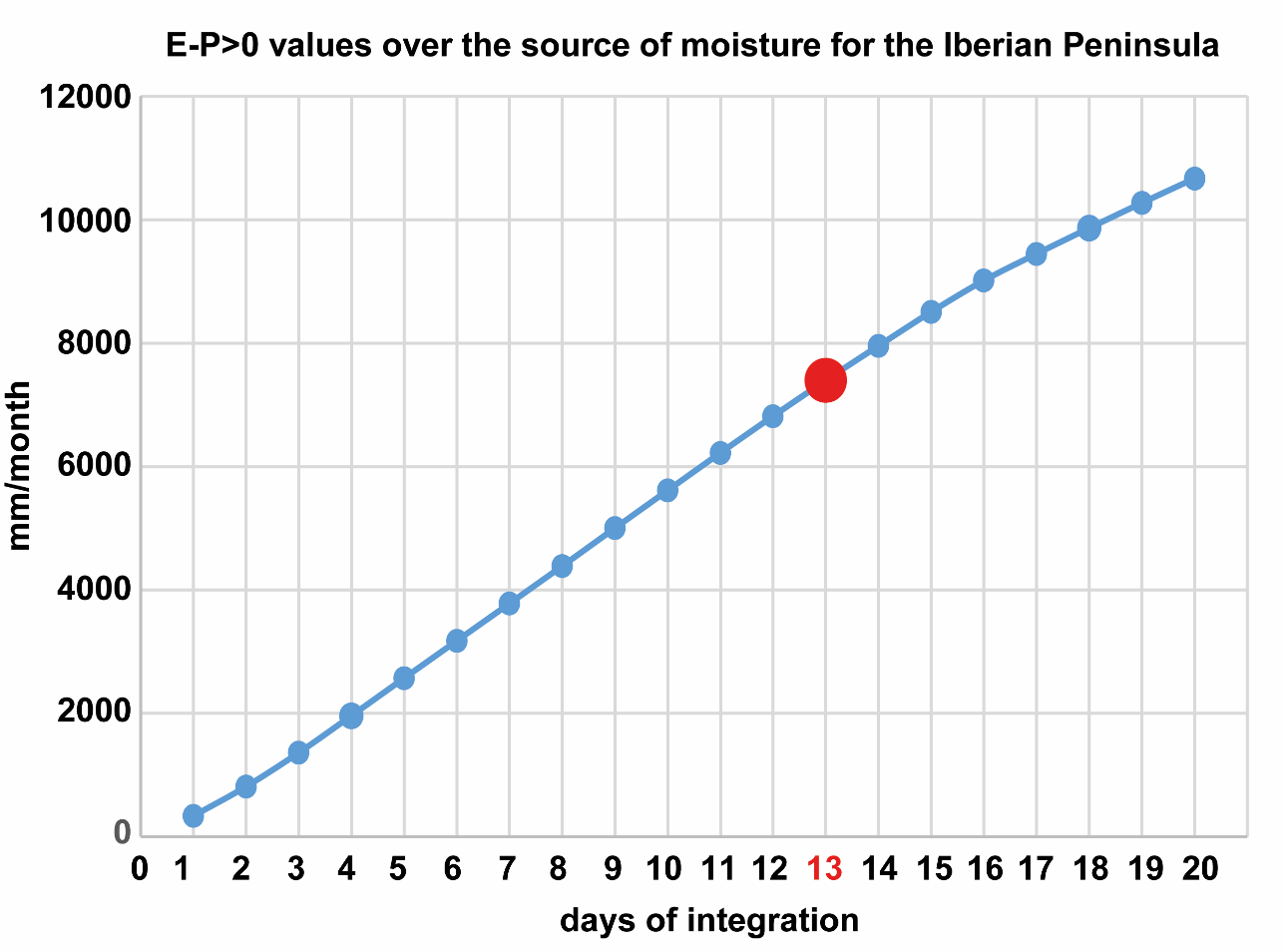


**Figure S3. Growing accumulated evaporation values over the moisture sources.** Accumulated (E − P)>0 values (evaporation) over each moisture source (Supplementary Figure S2) for every integration times for January for the Iberian Peninsula based on the backward in time experiment during 20 days. Period: 1980–2016. Highlighted with a red dot is the mean optimal time (i13) obtained for January over the region under study.


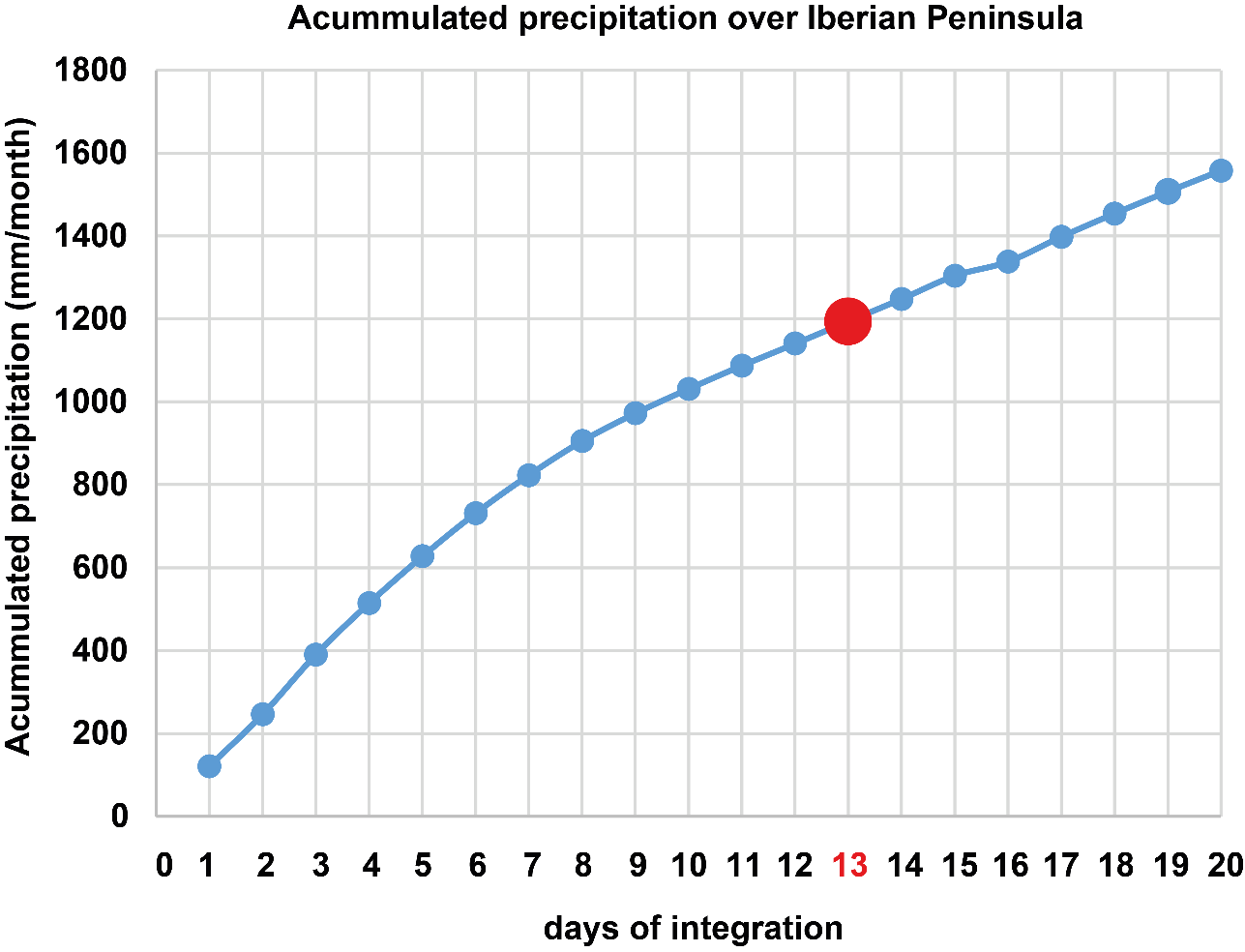


**Figure S4. Accumulated precipitation over the Iberian Peninsula along different days of integration.** Accumulated (E − P)<0 values (precipitation) over the Iberian Peninsula for January from each moisture source (from Figure S1) for every integration times based on the forward experiment during 20 days. Period: 1980–2016. Highlighted with a red dot is the mean optimal time (i13) obtained for January over the region under study.

**
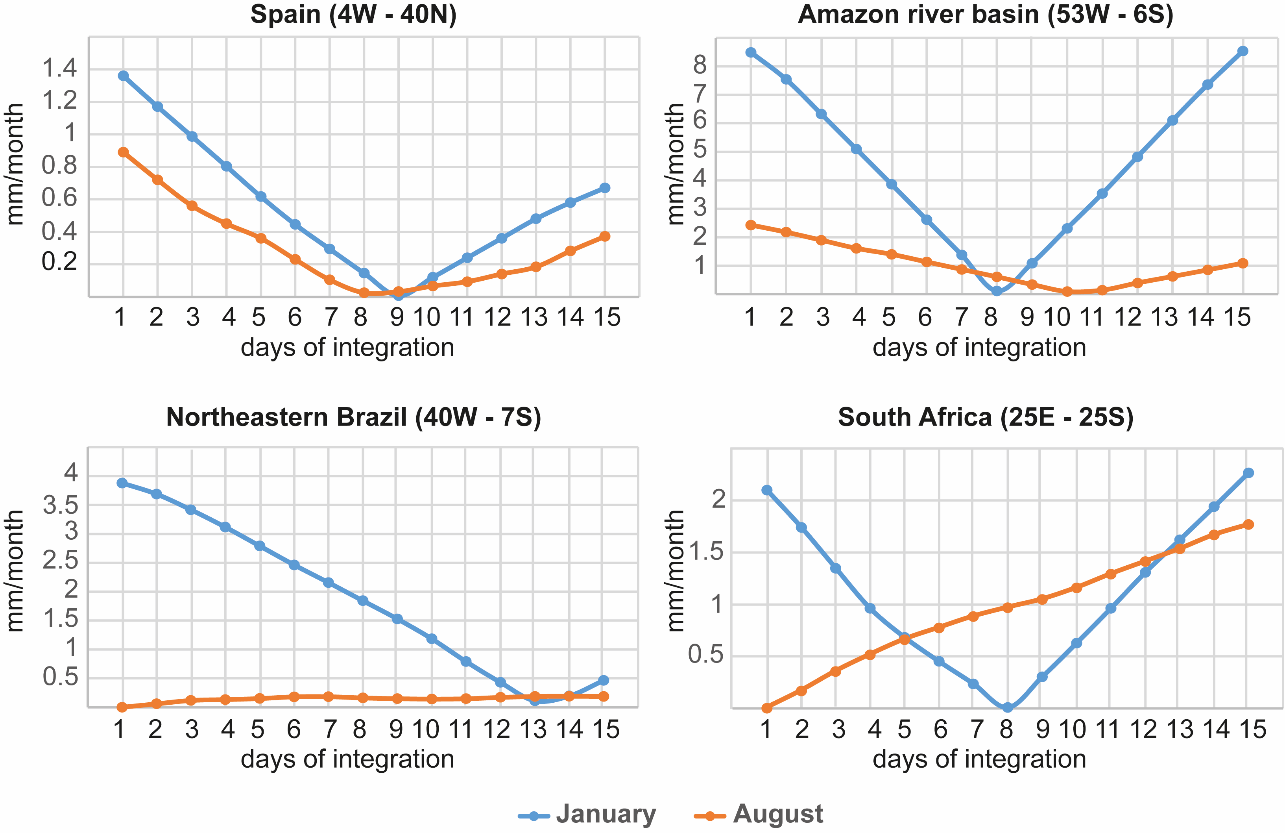
**

**Figure S5. Precipitation values for different day of integration.** Example of the precipitation values during the different days of integration for four grid points randomly selected for January and August.
